# Unintentional Underfuelling and Protein Prioritisation: A Multi-Methods Exploration of Nutrition Practices and Behaviours in Female Endurance Athletes

**DOI:** 10.3390/nu17233773

**Published:** 2025-11-30

**Authors:** Harvey O. Fortis, Colum J. Cronin, Kelsie O. Johnson, Sam O. Shepherd, Anthony C. Hackney, Juliette A. Strauss

**Affiliations:** 1 Research Institute for Sport and Exercise Sciences (RISES), Liverpool John Moores University, Byrom Street, Liverpool L3 3AF, UK; c.j.cronin@ljmu.ac.uk (C.J.C.); k.o.johnson@ljmu.ac.uk (K.O.J.); j.a.strauss@ljmu.ac.uk (J.A.S.); 2 Precision Fuel and Hydration Ltd., Christchurch BH23 7DX, UK; ss@precisionhydration.com; 3 Department of Exercise and Sport Science, University of North Carolina at Chapel Hill, Chapel Hill, NC 27599-8700, USA; ach@email.unc.edu; 4 Department of Nutrition, University of North Carolina at Chapel Hill, Chapel Hill, NC 27599-7400, USA

**Keywords:** female athletes, endurance, carbohydrate intake, protein intake, energy availability, nutrition practices, dietary behaviour, sports nutrition

## Abstract

Background/Objectives: Despite increasing awareness of best sports nutrition practices, discrepancies persist between knowledge and behaviour amongst female endurance athletes. Methods: To understand this discrepancy study investigated dietary practices, macronutrient intakes, and influ-encing factors using a multi-method approach. Seventy-two female endurance athletes (42 ± 9 y) completed four-day weighed food diaries, and a subset of twenty athletes (40 ± 10 y) then participated in semi-structured interviews. Quantitative analysis revealed that athletes met the lower end of carbohydrate (CHO) guidelines on rest days (3.0 g·kg^−1^), but intake fell short on training days, with deficits increasing as training volume rose (moderate: −1.4 g·kg^−1^, high: −3.5 g·kg^−1^, very high: −5.5 g·kg^−1^). Despite awareness of CHO’s role in performance, athletes unintentionally underfuelled, leading to a cumu-lative energy deficit. Energy intake increased by 473 kcal·day^−1^ per 1000 kcal·day^−1^ of exercise energy expenditure. In contrast, protein intake was prioritised, with mean in-takes of 1.7 ± 0.7 g·kg^−1^·day^−1^ aligning with recommendations. Results: Qualitative findings iden-tified barriers to CHO intake, including time constraints, diet culture influences and body image concerns. Social and environmental factors, such as household environments and professional nutrition guidance, played a critical role in behaviours. Conclusions: These findings highlight the need for practical, evidence-based nutrition interventions to support fe-male endurance athletes. Personalised education addressing CHO requirements, the psychology/emotions around nutrition, and the influence of social environments may bridge the gap between knowledge and practice, optimising both performance and long-term health outcomes.

## 1. Introduction

Over the past 50 years, female athlete participation has increased, with the Paris Olympics in 2024 being the first gender-balanced games [1]. However, the volume of research focused on the female athlete does not reflect this increased participation. For instance, carbohydrate (CHO) intake is well-established as a means of supporting training and competition demands [2]; however, as highlighted in a recent audit of 937 research studies which examined CHO intake recommendations [3], 11% (197/937 studies) of the participants in these studies were female. This raises concerns over the applicability of current evidence to women, which is largely extrapolated from data collected in males.

Maintaining energy balance (EB) appears to be a challenge for athletes with high training volumes. Studies indicate that negative EB is common among female athletes, particularly during periods of high training volume [4]. From a nutritional perspective, one major contributor to inadequate energy intake (EI) appears to be insufficient CHO intake, which is shown to be relatively low in female athletes, when compared to current recommendations. Studies have revealed that 50% of female soccer players and 85% of collegiate runners do not meet the daily CHO intake guidelines [5,6]. This trend is present in multiple ages and abilities, demonstrated by a study of young and elite female athletes, in which they report the prevalence of CHO intake below recommendations (<4 g·kg^−1^ BW·day^−1^) to be 49.2% in young female athletes and 33.3% in elite female athletes [7]. Further, the prevalence of CHO intake below the upper end of daily recommendations (<8 g·kg^−1^ BW·day^−1^) was 98.3% in young female athletes and 83.3% in elite female athletes [7]. Recently, it has been shown that knowledge of CHO guidelines does not correlate with CHO intake [8]. Using the CHO for endurance athletes in competition questionnaire (CEAC-Q), a third of athletes correctly identify the CHO loading and pre-competition CHO recommendations, whilst two thirds of the athletes (*n* = 32, 64%) were able to identify the CHO guidelines for during exercise. Despite this, 32% of participants consumed the recommended CHO for events lasting >2.5 h in duration (60–90 g·h^−1^), with two athletes consuming >90 g·h^−1^. In contrast, suboptimal CHO intakes during competition were observed in 64% of athletes, with CHO intake for eight athletes (16%) classified as very low (<30 g·h^−1^) [8]. This suggests there is a disconnect between knowledge and application of nutrition guidelines. One recent study explored elite female soccer players’ perceptions of nutrition, specifically CHO, and highlighted confusion and misconceptions as key drivers to under-consumption of CHO. This study revealed that these behaviours appear to stem from external pressures, social media, and the implementation of body composition testing [9]. This fear of CHO is suggested to lead to intentional CHO restriction, despite CHO-fuelling strategies being well-documented as beneficial for health and sports performance [9]. However, the applicability of these findings to endurance athletes are not known and this remains an unexplored area of research.

Beyond CHO, there is a general lack of understanding around other nutrient requirements for female athletes. For instance, much of the research around protein is conducted in male athletes, where protein plays a vital role in muscle repair, synthesis, and recovery following endurance exercise [10]. Whilst it has previously been shown that females are more likely to under-consume protein [11,12], the reasons behind these lower intakes are not fully detailed, and habitual protein habits are not reported. Holtzman et al. [13], highlight the importance of adequate protein intake among female athletes, emphasising its role in promoting lean muscle mass maintenance and optimising training adaptations. Despite the recognition of protein’s benefits, little research has been conducted in female athletes, and there is currently no evidence to suggest that there are any sex differences in protein requirements [14]. Fat is also a vital nutrient for athletes, playing a crucial role in energy production, hormone regulation, and cellular function. For athletes, fat provides a sustained energy source, especially during prolonged endurance activities, and is also essential for the absorption of fat-soluble vitamins (A, D, E, K) and for maintaining cell membranes and nerve function. Female athletes’ current habits and behaviours around fat intake are not well-explored, but some insights suggest that female athletes could be at risk of under consuming fat, especially in sports where leanness is prioritised [15].

Endurance athletes’ macronutrient requirements vary according to training status, session duration, and intensity. CHO remains the principal substrate supporting moderate to high-intensity endurance exercise, with daily needs typically ranging from 3–5 g·kg^−1^·day^−1^ during low-volume phases to 8–12 g·kg^−1^·day^−1^ during periods of heavy load or competition [2,16,17]. Protein requirements may fluctuate to a lesser extent, increasing modestly during phases focused on strength development or body-composition changes, while adequate dietary fat supports energy provision and endocrine function. These demands also vary within and between days as training intensity and recovery needs shift [18]. While endurance disciplines such as triathlon, cycling, and running differ in training volume, session structure, and logistical demands, their underlying principles remain similar: each presents the overarching challenge of matching fluctuating energy expenditure (EE) with sufficient nutritional intake [19,20].

Among female athletes, the effect of hormonal fluctuations across the menstrual cycle on fuelling and hydration strategies is often reported as a topic of interest. Current evidence suggests these variations may influence nutrition subtly, through thermoregulation, gastrointestinal comfort, and fluid balance, with potential behavioural implications for food choice and appetite, rather than large shifts in substrate requirements per se [21,22]. Beyond physiological influences, psychosocial factors have emerged as powerful determinants of female athletes’ nutrition behaviour. Research increasingly highlights the influence of diet culture, body image, and social comparison, often amplified through social media, as drivers of restrained eating, CHO avoidance, and “clean eating” ideologies [9,23,24]. Such factors may contribute to transient periods of low energy availability (LEA) and, in some cases, to the broader syndrome of relative energy deficiency in sport (RED-S). However, the aetiology of RED-S is multifactorial, and EI alone rarely explains the full picture; psychological stress, training load, and recovery practices also play important roles [25,26]. Chronic mismatches between EI and expenditure can negatively influence bone turnover, hormonal regulation, and training adaptation. Yet despite growing recognition of these issues, the long-term consequences of underfuelling and macronutrient imbalance remain under-researched, particularly in women.

The dietary behaviours of female endurance athletes are multifaceted and influenced by a combination of physiological, psychological, social, and environmental factors. Addressing these complex interactions is essential to implement support and guidelines for this population. It is important to understand the real-world challenges and perspectives of female athletes to develop effective interventions. Accordingly, this study aims to comprehensively investigate the nutrition practices, habits, and perceptions of female endurance athletes using a multi-methods approach. Quantitative assessments will provide insights into macronutrient intake, EE, and nutrient timing. The qualitative interviews will explore athletes’ experiences and perceptions, and aim to understand contextual factors that influence dietary behaviours. The integration of multi-methods seeks to elucidate the complex interplay between nutritional intake, psychological factors, social influences, and athletic performance in female endurance athletes. A degree of underfuelling, with CHO intake falling below recommendations, particularly amidst greater training demands, was hypothesised. Protein intake was also anticipated to be inconsistent, with athletes likely failing to meet or distribute recommended amounts. Finally, we hypothesised behaviours to be shaped by a combination of practical and psychosocial factors, reflecting the complex interplay between knowledge, behaviour, and real-world contexts.

## 2. Materials and Methods

### 2.1. Study Design

A multi-method design, combining qualitative and quantitative methodologies, was implemented to understand female endurance athletes’ dietary habits and behaviours, as well as their perspectives and experiences. This study employed triangulation and a convergent mixed methods design, guided by the pragmatic approach outlined by Bishop [27]. The convergent design involves simultaneous collection and analysis of quantitative and qualitative data streams, allowing for comparisons or relations to be made between data sets. Both data types were given equal priority, reflecting a commitment to a balanced multi-methods approach. Quantitative and qualitative results were integrated during the interpretation phase to address the research question holistically and derive nuanced insights.

Athletes completed an initial questionnaire to provide personal information and to ensure their eligibility to participate. Following this, athletes completed a 4-day dietary assessment via the remote food photography method (outlined below). Finally, a subset of athletes attended follow-up individual interviews to discuss their nutrition perspectives in greater detail. Institutional ethical approval was granted by the LJMU Research Ethics Committee, UREC reference: 23/SPS/035.

In line with the adopted convergent design [27], quantitative methods are hereby presented first before the qualitative methods. This is followed by outlining the ‘mixing’ process of the data.

### 2.2. Quantitative Methodology

#### Participants

To meet the studies inclusion criteria, all participants (*n* = 72) were >18 years old, actively participated in >7 h per week of various combinations of sports which contribute to an endurance training programme (running, cycling, swimming or triathlon), had regularly trained in endurance sports for over 2 years, and had no known diagnosed eating disorders. Participants’ training volumes differed from 7 to >15 h per week (see Table 1). In addition to endurance sports, some athletes reported that they perform yoga (*n* = 16) and strength training (*n* = 31) to supplement their endurance programmes. To recruit this sample, participants were contacted through various social media platforms to encourage participation of a wide range of individuals, with different backgrounds and geographical locations. Nevertheless, recruitment posters were also sent to relevant locations, such as university buildings, local sports centres, and training facilities, and discussions with endurance training clubs were held to provide details of the study and participant information.

Together, this sample provided the generation of a rich data set to support an in-depth understanding of nutrition in sub-elite female endurance athletes, categorised as tier 2–3 level athletes [28]. All participants provided verbal and written informed consent before completing the food diaries and interviews. With the depth of findings in mind, we embraced the relatively new concept of informational power to guide the sample size, whereby in-depth rich insights are valued over volume data sets [29].

### 2.3. Quantification of Dietary Intake

**Figure 1:**
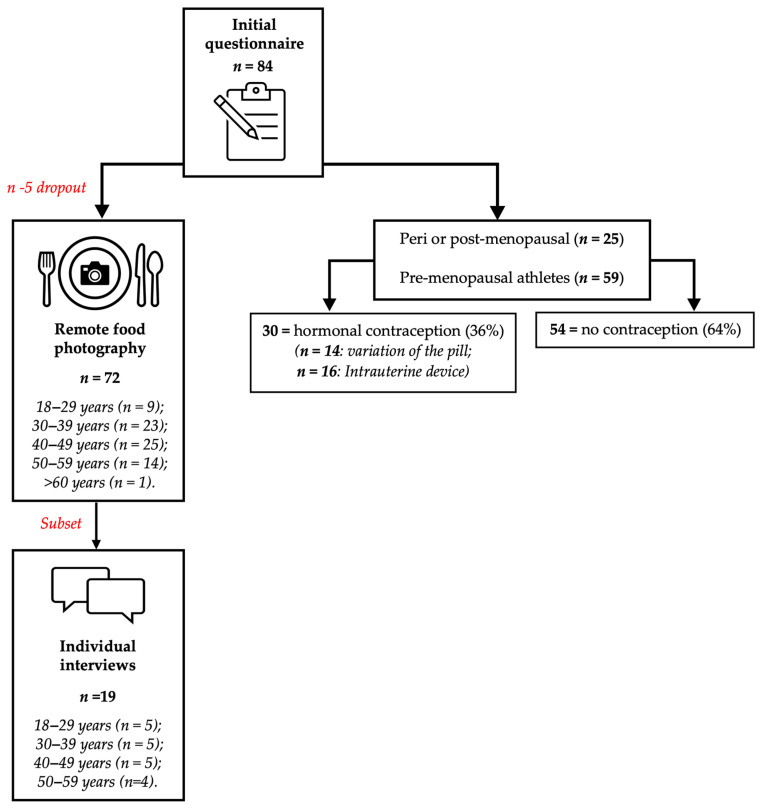
Flow chart of participation process from initial questionnaire responses to remote food photography data collection, to individual interviews. Of the 84 athletes who initially expressed interest, 72 completed the quantitative component. Nineteen participants were subsequently interviewed as a subset sample for interviews and qualitative analysis. Red text demonstrates the particpaton during different phases of the study.

Of the initial questionnaire (*n* = 84), 72 athletes completed a follow-up food diary, with 12 participants withdrawn from the study due to incomplete food diaries. A flow chart, demonstrates the participation numbers throughout different phases of the study (see Figure 1). Self-reported energy and macronutrient intakes were assessed across 4 days using a modified version of the remote food photography method (RFPM), a method shown to accurately measure the EI of free-living individuals [30], including athletes [31]. The use of photographs helps mitigate common reporting biases, such as underreporting or overreporting of food intake, by providing a visual record that can be cross-verified [32]. Remote submission allows for real-time data collection, enabling prompt feedback and reducing the likelihood of participants forgetting to record their intake [33]. Participants were trained on how to accurately weigh and photograph their food. This included instructions on using digital scales to measure food portions and taking clear, consistent photographs of their meals before and after consumption. Participants were sent a training video (11 min 58 s) to provide clear instructions around the best practices of the method. Participants were then asked to demonstrate that they had engaged with and retained the information, with a short follow-up quiz. Athletes were given the opportunity to ask any questions prior to data collection and were encouraged to wait at least 2 days after watching the video prior to starting the RFPM, to allow them time to become familiarised with the protocol and ask questions. In brief, athletes provided a high-resolution photograph and description of all their food and drinks before and after consumption. Photographs were timestamped alongside a written or spoken (voice note) description of the food/drink (including information on quantities, brands, preparation, and cooking methods) and a known-size visual reference (e.g., credit card or ruler) and then submitted to an app-based chat (WhatsApp, Dublin, Ireland) with the principal researcher. To ensure athletes did not exclude any foods/drinks and increase the accuracy of food records, researchers sent a daily reminder the morning of each day and prompted the athletes for further information, where necessary (e.g., if a photograph contained items that were difficult to identify). To limit bias or influence, researcher feedback was restricted during the 4-day data collection period. The only communication was to request for further details (such as food weight) and to answer any questions athletes had regarding the method. Dietary intake was analysed individually, on two separate occasions, by a trained and experienced member of the research team (>2 years’ experience as a registered nutritionist), using dietary analysis software (NutriticsTM version 6.14, Dublin, Ireland), which calculated energy and macronutrient intake for each athlete. A secondary practitioner checked all inputs independently and randomly re-analysed 25% of food diaries, to enhance scientific rigour. Output values from the two analyses were averaged to provide estimates of EI in kilocalories per day (kcal·day^−1^) and macronutrient intakes, reported in grams (g) and grams per kg body mass (BM) (g·kg^−1^). Daily dietary records were analysed at the individual day level, as independent observations, reflecting the study’s aim to characterise free-living fuelling behaviour under different training loads, rather than to evaluate repeated measures change within participants. Because no within-participant hypotheses were tested, simple linear regressions were used to quantify associations, which should be interpreted descriptively rather than causally. To further assess athletes’ nutrition behaviours, dietary intake data was also stratified into four categories dependent on daily training volume, based upon the current consensus of CHO requirements for training load [16]. Based upon these guidelines, data were grouped into intakes from: rest days (<45 min exercise), moderate training days (45–90 min exercise), high training days (90–210 min exercise) or very high training days (>210 min exercise). Data analysis also explored different days of the week, by observing individual days and weekdays to weekend days, where typically, training volumes may differ.

### 2.4. Exercise Energy Expenditure

Metabolic EE was estimated from mechanical work values recorded from the participant’s power metres (Mechanical Work (kJ) = Power (W) × Time (s) ÷ 1000. Metabolic EE (kcal) = Mechanical Work (kJ)/GE × 0.239), using validated methodology in female cyclists [34]. Whilst acknowledging the existence of inter-individual variation in athletes, an estimated gross efficiency (GE) of 20% was used for all athletes for the calculation of metabolic EE. This GE value was based upon the assumption that average GE would be equal to 20%, in line with reported values from similar populations [35,36]. Although GE varies between individuals (typically ~18–25%) depending on intensity, cadence, and training status, use of a standardised value ensures comparability with the prior literature and across participants. To examine robustness, we conducted a sensitivity check by re-expressing exercise energy expenditure (EEE) over GE = 18% and 22% (i.e., EEE_18_ = EEE_20_ × 1.111; EEE_22_ = EEE_20_ × 0.909) and compared associations with EI [35,36,37,38]. For non-cycling sessions, EEE was estimated using heart rate (HR) data, as a validated and practical tool for remote data collection [39,40,41,42,43]. Participants were required to use validated devices capable of continuous HR recording (e.g., Polar H10, Garmin HRM-Pro, or COROS HR Armband). Wrist-based optical HR sensors were not permitted due to their lower accuracy during movement [43,44]. These estimates are based on the observed linear relationship between HR and oxygen consumption (VO_2_) during steady-state aerobic exercise, enabling indirect calorimetry-based validation. For example, Keytel et al. [39] developed equations that show strong correlations between HR-derived EE and values measured via indirect calorimetry (*r* = 0.73–0.95) during aerobic activities. Similarly, Hiilloskorpi et al. [40] demonstrated the reliability of HR monitoring combined with individual calibration for estimating EE in recreational and trained individuals, reporting an average error of ~10% during steady-state activities. However, potential error propagation may occur when estimating total daily EE, particularly during intermittent or high-intensity exercise where HR–VO_2_ coupling is reduced.

Recent advancements have explored HR-derived EE for remote data collection in wearables. Montoye et al. [41] found that while wearables provided reasonable EE estimates for walking and running, accuracy declined during non-aerobic activities, highlighting the limitations of HR-derived EE in mixed-mode activities. Similarly, a systematic review by Fuller et al. [42] found that integrating physiological sensors like heart rate with accelerometery improved EE estimates, but accuracy varied depending on activity type. Research-grade devices outperformed commercial ones for total EE but struggled with ambulatory and sedentary tasks, emphasising the need for further refinement in wearable EE estimation.

### 2.5. Qualitative Methods

To explore athletes’ perceptions and personal contexts, we took a qualitative approach. Qualitative research provides a route to understand the experiences and perceptions of individuals who function as a part of a complex, multifaceted environment [29]. As such, it was deemed an appropriate methodology for the present study.

### 2.6. Participants

To gain detailed insights into the perceptions of nutrition in our population of interest, a subset (*n* = 19) of participants from the quantitative component of this study were purposefully invited to take part in follow-up interviews. This approach is comparable to previous qualitative explorations of nutrition practices in sport [9,45,46]. The inclusion criteria for the purposeful sample match the inclusion criteria for the quantitative component of this study. To recruit this sample, participants could optionally opt in to the qualitative component of the study, with the understanding that this did not guarantee their participation, with a random subgroup being selected for the qualitative component. Due to the broad range of participant ages, randomisation was applied to decade age groups, to encapsulate the breadth of ages in the qualitative data.

### 2.7. Data Collection

Semi-structured interviews (43 ± 12 min) were conducted, with all participants using online video conferencing software (v.2025.2.5.1, Microsoft Teams), and were audio-recorded. Interviews were conducted in private rooms, with headphones, providing an accessible and safe space for participants to share their experiences and perceptions. The interview was centred on understanding the participants’ nutritional perceptions and influences. The semi-structured questions were designed based on the primary study aims; however, it was challenging to develop questions on previous research and theory due to a lack of qualitative studies conducted in this population [47]. Accordingly, an inductive approach was undertaken, where the interviewer aimed to explore athletes’ experiences, influences, and perspectives on several areas, (1) ‘Life experiences’, (2) ‘current day to day life’ influences, and (3) ‘training and competition’. Consistent with this ‘open-ended’ [48] interview format, all questions were discussed in a free flowing conversational and informal manner, to allow for maximum voluntary contribution and detail. For example, questions began with phrases such as, ‘What are your thoughts on…?’ and ‘How would you…?’. Following this, naturally occurring probing questions [49] were asked to gain further detail, where necessary. This format of investigation allowed participants the opportunity to share their experiences and perspectives with minimal influence from the researcher and encouraged self-driven conversations [50]. Data collection was ceased when saturation was determined. Given the study’s focus on obtaining depth rather than breadth of perspectives, recruitment continued until rich, detailed accounts were achieved and no substantially new concepts emerged. Once recurring insights were observed across participants, data collection was considered complete.

### 2.8. Analysis

All interviews were transcribed using Microsoft Teams and later manually refined to ensure scripts were verbatim. The inductive approach was maintained, to place the voices of the participants at the forefront of the analysis. The lead author identified meaningful segments of text based on the question domains. As part of the analysis process, these meaningful segments were subject to initial (open) coding [51]. Once this initial coding was complete, codes were revisited as part of a focused process to identify potential themes across the data, and to consider the research aim. Themes were subsequently developed over several iterations by the lead researcher. Through discussion with the research team, these themes were refined to provide a credible and trustworthy ‘common thread’ [52], which is presented in the findings to come.

### 2.9. Rigour

Several procedures were performed to ensure credible and transparent perceptions of female endurance athletes’ experiences and perceptions on nutrition. These procedures are utilised as a method to add rigour whilst maintaining focus on the athletes’ individual and subjective understanding and experiences, an important component of qualitative research [53,54]. For example, interviews were conducted by a researcher trained in qualitative methods and experienced as a nutritionist and athlete in endurance sports. Aware of individual subjectivities, interview questions were examined prior to data collection by a critical friend to ensure that they were not leading. Pilot interviews were conducted to develop questioning and follow-up probes, so that they were appropriate and neutral. Multiple critical friends [55] were used, with a range of expertise and experiences to check and challenge data analysis, theme generation, and the presentation of selected quotes that arose. The critical friends included individuals with no experience in nutrition, but expertise in qualitative research and an individual with no experience in qualitative research, but expertise in nutrition in female endurance sports. The role of the critical friends is ‘not to “agree” or achieve consensus, rather to encourage reflexivity by challenging each other’s construction of knowledge [55,56]. Consistent with this, the critical friend challenged the coding process, and as recommended [38], themes were refined over time to provide a trustworthy account of participants’ experiences.

### 2.10. Mixing of Data

The mixing of data was performed by comparing the findings between data sets. Quantitative data was objectively analysed with reference to research guidelines, which in turn was used to target focused analysis of interviews to compare objective measures to athletes’ own beliefs and interpretations. Both sets of data streams were analysed and interpreted before the data was combined. Data was accumulated and interpreted by the lead researcher, before presenting to the wider research team, undergoing a rigorous ‘critical friend’ process. As recommended by Smith and McGannon [54], data was presented to individuals external to the research team, including research participants, who confirmed that the outcomes were representative of the data.

### 2.11. Reflexivity

In line with qualitative research best practice, reflexivity was maintained throughout data collection and analysis to acknowledge the researcher’s influence on interpretation [52,57]. The lead author is both a nutrition practitioner and participates in endurance sports, bringing a contextual understanding, but also potential for interpretive bias. To mitigate this, reflexive journaling was maintained during coding and theme development, documenting assumptions, emotional responses, and decision-making [58]. Critical friends with expertise in social science, psychology, and physiology were engaged throughout the process to check and challenge interpretations, offer their positional insights, and ensure transparency [54]. This triangulation of perspectives fostered reflexivity and strengthened the trustworthiness of findings by encouraging alternative viewpoints rather than consensus.

## 3. Results

In keeping with Bishop et al.’s (2015) convergent mixed design method [27], quantitative data will be presented, followed by qualitative data. Data has then been subsequently mixed to form interpretations via a combined discussion.

### 3.1. Quantitative Results

**Figure 2:**
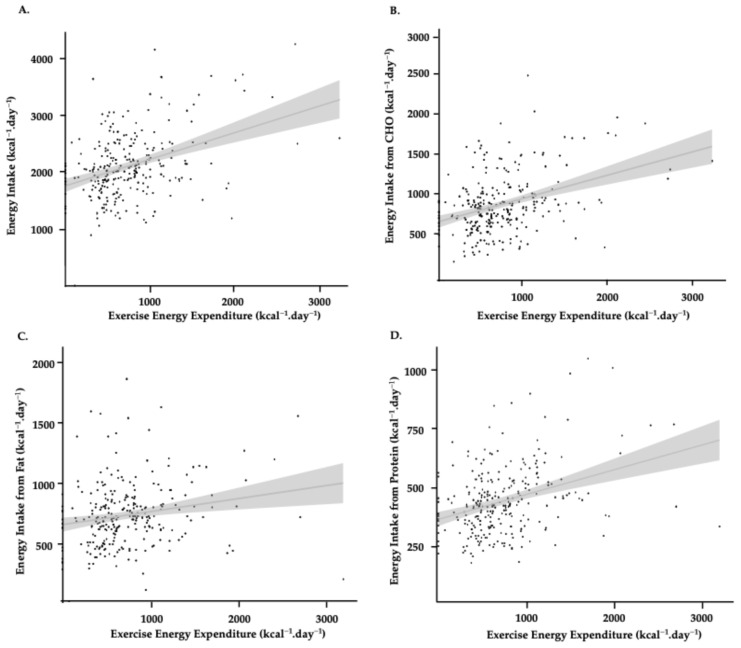
Relationships across the 4-day monitoring period between EEE and energy intake (**A**), energy intake from CHO (**B**), energy intake from fat (**C**), and energy intake from protein (**D**), all expressed to kcal·day^−1^. Individual dots represent individual data points for each day of food tracking. The grey line signifies the line of best fit from a simple linear regression model, and the shaded grey represents SD.

**Figure 3:**
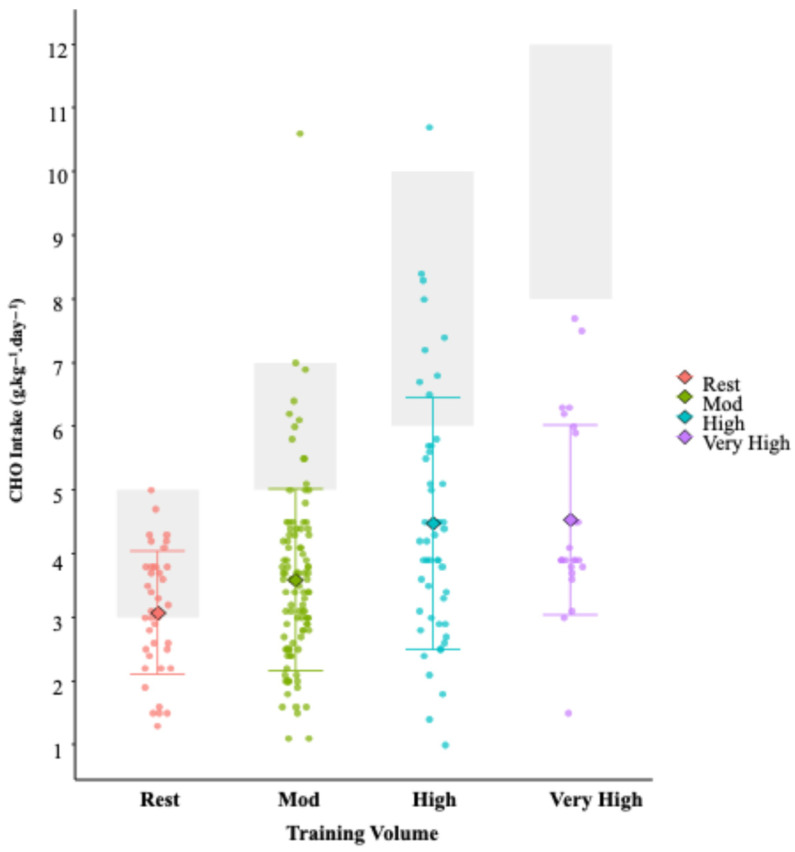
Relative CHO intake of the cohort of female endurance athletes, stratified by daily training volume (Rest: <45 min training; Mod (moderate): 45–90 min training; High 90–210 min training; V. High (Very High): >210 min training). Shaded grey boxes denote CHO intake recommendations that correspond to the daily training load, according to the guidelines of Thomas et al. [16]. Individual dots represent daily values; central diamonds indicate averages, and error bars show standard deviations.

CHO, fat, and protein accounted for 43.1 ± 11.0, 33.3 ± 10.1, and 20.4 ± 6.4% of daily EI, respectively, throughout the assessment period. In relative terms, CHO, fat, and protein intakes were 3.8 ± 1.6, 1.3 ± 0.5, and 1.7 ± 0.7 g·kg^−1^·day^−1^, respectively, (Table 2). We found a moderate positive within participant relationship between EEE and EI (*r* = 0.38; 95% CI 0.27–0.48, *p* < 0.001; Slope = 0.47) (r = 0.39; 95% CI 0.29–0.49, *p* < 0.001; Slope = 0.29). This translates into an increased energy intake of 473 kcal·day^−1^, for every 1000 kcal·day^−1^ increase in EEE (see Figure 2). Total EI increases in total daily EI from rest (1788 kcal), to moderate (2004 kcal), to high (2337 kcal), to very high (2542 kcal) training days (see Figure 3). Whilst athletes appear to partially compensate for increasing EEE by increasing their EI from CHO, on most days they failed to fall within the recommended CHO intake [16] (Figure 3). This is reflected in under-consumption of CHO on 50% of “Rest” days, 86% of “Moderate”, 93% of “High”, and 92% of “Very High” volume training days. The magnitude by which CHO practices fail to meet the lower end of their respective guideline range increases from ‘moderate’ (−1.4 g.kg^−1^ BM), to ‘high’ (−3.5 g.kg^−1^ BM), to ‘very high’ (−5.5 g.kg^−1^ BM).

We found a moderate positive within-participant relationship between EEE and EI from CHO (*r* = 0.39; 95% CI 0.29–0.49, *p* < 0.001; Slope = 0.29). (see Figure 2). This translates into an increased EI from CHO of 292 kcal·day^−1^, for every 1000 kcal·day^−1^ increase in EEE. We found a weak but statistically significant positive relationship between EEE and EI from protein (*r* = 0.35; 95% CI 0.24–0.45, *p* < 0.001; Slope = 0.11) (see Figure 2). Although significant, the small slope indicates that increases in protein intake were modest relative to changes in EEE. Lastly, we also found no relationship between EEE and EI from fat (r = 0.19; 95% CI 0.07–0.30, *p* < 0.001; Slope = 0. 0.11) (see Figure 2). Results were robust to reasonable variation in GE. Re-expressing EEE using 18% and 22% GE left correlations unchanged (EI vs. EEE: *r* = 0.39; CHO vs. EEE: *r* = 0.38; all *p* < 0.001) and altered the translation per +1000 kcal EEE by approximately ±10% (EI: ~423–517 kcal; CHO: ~261–319 kcal) relative to the 20% baseline (473 kcal and 292 kcal, respectively). Although these differences (< 100 kcal·day^−1^) are small in absolute terms, they may still hold practical relevance when interpreting individual-level EB, particularly in athletes with high training volumes or tightly controlled energy targets.

Across the full sample, mean daily energy intake was 2107.7 ± 611.1 kcal, while mean daily energy expenditure was 2289.0 ± 557.1 kcal, resulting in an average daily energy deficit of −193.4 ± 576.3 kcal and a cumulative 4-day deficit of −772.1 ± 1523.3 kcal (Table 3). When assessing for day of the week differences, EB for weekdays was −119.4 ± 499.5 kcal and for weekend days was −420.3 ± 743.1 kcal (see Table 4). Individually daily EB are also presented for each day of the week in Table 4.

Energy balance was negative across all age categories, ranging from −120.1 ± 217.1 kcal·day^−1^ in athletes aged 50–59 years to −482.2 ± 71.0 kcal·day^−1^ in those aged 18–29 years, with the single participant aged ≥60 years reporting −768.4 kcal·day^−1^ (see Table 5).

### 3.2. Qualitative Results

**Figure 4:**
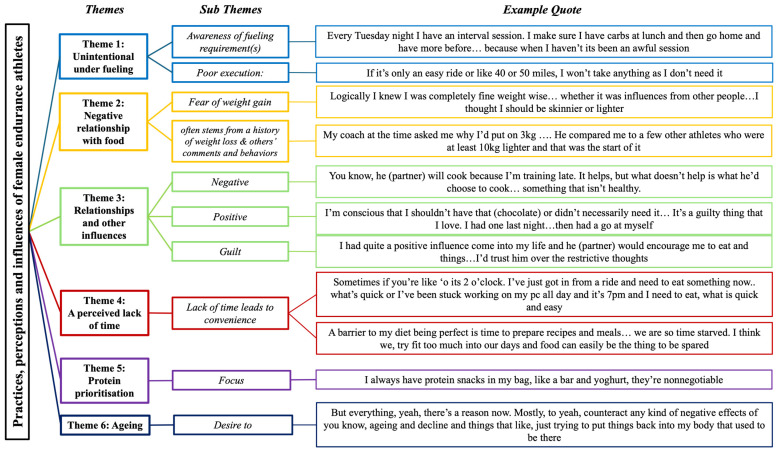
Assimilation of themes and example quotes from individual interviews.

Following data synthesis and analysis of the interview transcripts, six themes were established that present a narrative of the nutrition practises of female endurance athletes (Figure 4). These themes are presented below, firstly in a thematic map *n*, which summarises qualitative findings, followed by each theme elaborated and presented in depth, with athletes’ quotes presented verbatim to support the narrative.

To further enhance transparency, participant characteristics contributing to each qualitative theme are summarised in Table 6. This table outlines the distribution of participants by age range and training volume, across the six themes. Several participants contributed to more than one theme, reflecting the interconnected and multifactorial nature of nutrition-related experiences among female endurance athletes.

#### 3.2.1. Theme 1: ‘Unintentional Underfuelling’

There appears to be a disconnect between female endurance athletes’ understanding of fuelling strategies and their execution. Participants demonstrated a conscious effort to fuel appropriately for high-intensity or long-duration sessions; however, they often showed complacency toward shorter or lower-intensity sessions. Although they acknowledged the increased nutritional demands of their training, their CHO intake did not consistently reflect this awareness. While participants expressed some understanding of the need to adjust fuelling based on training load, their reported behaviours frequently did not align.

Awareness of the importance of adequate fuelling are demonstrated here:

Every Tuesday night I have an interval session. I make sure I have carbs at lunch and then go home and have more before as well, because when I haven’t’, it’s been an awful event, an awful session.

(Participant 1)

I can’t afford to dig a hole today, because that’ll put me in a hole tomorrow and maybe the next day.

(Participant 5)

Evidence of suboptimal application of the nutrition requirements for training demands:

I haven’t done any sessions lately that are long enough to need nutrition. Like only if it gets over 2 h is when I think about nutrition.

(Participant 8)

If it’s only an easy ride or like 40 or 50 miles, I won’t take anything as I don’t need it.

(Participant 4)

This gap between understanding and practice, highlighted in interview responses, was underscored by athletes’ frequent use of the word “only” when referring to training sessions, suggesting a minimisation of the sessions’ nutritional demands. Quantitative data further reinforces the mismatch between female endurance athletes’ perceived and actual CHO intake. Across all training days, ranging from moderate (−1.4 g·kg^−1^ BM) to high (−3.5 g·kg^−1^ BM) and very high (−5.5 g·kg^−1^ BM) training volumes, participants consistently failed to meet established CHO recommendations.

#### 3.2.2. Theme 2: Negative Relationship with Food

Many female athletes in endurance sports fear future weight gain and are impacted by past experiences and negative thoughts around food. These individuals in the present study experience daily thoughts (sometimes their subconscious) and inner monologues around food, thereby impacting their day-to-day food behaviours.

I was in a healthy range on the scale of what is a healthy BMI… logically I knew I was completely fine weight wise, but I still like, whether it was influences from other people and that kind of stuff, I think I thought I should be skinnier or lighter.

(Participant 5)

Especially as a female, like the pressure, because like realistically, if someone looks at me, I don’t look like a stereotypical endurance athlete, because I’m naturally quite muscular, so I am naturally heavier anyway. Like when I was lean and had a six pack, I was still like 62/63 kg and that was the lightest I ever was and I think that for me, was unhealthy. Like normal weight is more like 65/66 kg, maybe a little more at the moment. Some of my closest friends like (names friends) are all like 55 to 60 range and I think people forget that with your specific body type, there’s only so much weight you can lose, like you can’t compare. I did compare myself to others and thought they’re lighter than me and perform well, so.

(Participant 63)

The fear of future weight gain often stems from a history of weight loss and past experiences with external comments and behaviours from others.

Someone called me fat in the street once and that made me lose weight very quickly.

(Participant 1)

My coach at the time asked me why I’d put on 3 kg over Christmas. He compared me to a few other athletes who were at least 10 kg lighter and that was the start of it.

(Participant 63)

Poor experiences and complicated relationships with food led some participants to negatively label certain foods and experience recurring feelings of guilt. Energy-dense items, commonly included in an endurance athlete’s diet, were often assigned negative connotations. This placed female endurance athletes in a difficult position, caught between the nutritional needs of their training and their emotional or moral judgments about food.

I’ve never been slim, and I have always been conscious about my weight, I have never really eaten freely without guilt, and it’s always been like if I am eating, it’s just like, oh no I shouldn’t be eating this or I am going to put on weight.

(Participant 21)

I suppose at times, yeah, I have bad days when I go, I am never going to get rid of these thoughts because they’re a part of like, who I am, they’re always going to be there, just to a lesser degree. But I can just handle it better and I have to tell myself, like you know what you’re doing, you know that even if you don’t think you should eat, you need to, like you know there’s science behind this and you know the logic.

(Participant 5)

Thus, Theme 2 demonstrates the deep-rooted psychological struggles that many female endurance athletes face when navigating food, weight, and body image. In essence, these athletes are not just managing the physical demands of their exercise, but also grappling with social pressures, historical comments, and ingrained food-related guilt.

#### 3.2.3. Theme 3: Relationships and Other Influences

Athletes’ personal ecosystems, such as partners, children, and support professionals, can significantly shape their dietary behaviours, both positively and negatively. For some sub-elite female endurance athletes in this study, external influences played a substantial role in daily food choices, often limiting autonomy and compromising nutritional quality. These athletes described situations where their food intake was dictated by the preferences, habits, or schedules of others around them, rather than by their own training needs.

I wouldn’t ever choose to have a takeaway, but he (partner) loves them. So, we will have one every two weeks or something and like. I like them, but I wouldn’t just have one, because I’d just cook.

(Participant 34)

You know like, he (partner) will have to cook because I’m training late. It helps, but what doesn’t help is what he’d choose to cook, like a pizza or something that isn’t healthy, and is not what I’d want.

(Participant 31)

They (children) need driving places and have lots of commitments, which dictates when I can eat.

(Participant 2)

In contrast, other athletes in this study reported positive influences that encouraged more supportive and health-focused eating behaviours. For these individuals, key figures, such as partners, dietitians, or nutritionists, played an important role in helping them improve their relationship with food and rebuild confidence in their eating decisions.

I had quite a positive influence come into my life, and he (partner) would encourage me to eat and things and because he knows what he’s talking about, I’d trust him over the restrictive thoughts I’d have.

(Participant 1)

I made peace with it, after developing a bad relationship with food scarily quickly. I worked with her (dietitian), who helped me remember how to eat properly.

(Participant 3)

Yeah like, the conversation in my head about food is completely different now and since working with a nutritionist, I am able to do things with less worry and be confident in what I choose to eat and things.

(Participant 2)

Thus, Theme 3 demonstrates how the social and domestic environment can either support or hinder athletes’ nutrition practices. The people around athletes can contribute as barriers or facilitators, influencing food behaviours through shared meals, time pressures, emotional support, or professional guidance.

#### 3.2.4. Theme 4: A Perceived Lack of Time

Demands on time and a lack of planning lead to athletes searching for convenient food or skipping meals. Balancing the time constraints of training and other commitments often leads to nutrition being compromised.

There’s a reason why I wouldn’t train and probably that I don’t have the time, so I’d get home late, too late to do a session or I’d have so much on and that would then constitute me not wanting to cook dinner, because well I can’t be bothered and I think ‘oh actually it’s okay because, I didn’t train so don’t need it anyway.

(Participant 3)

Yeah, I hate that (planning). It’s just really hard work and that sounds ridiculous because I plan everything else, like work and training. I could plan to make a meal on, like, a Tuesday night, and when it comes to it, I just can’t be bothered. I really don’t like thinking about food, it stresses me out.

(Participant 1)

A barrier to my diet and that being perfect is time to prepare recipes and meals, so I’m better off having a pack of microwavable rice and veggies and things that are super simple, because we are so time starved. I think we, like a lot of people, try fit too much into our days and spare time isn’t really a thing, so food can easily be the thing to be spared thought or prep. We set ourselves up to fail every day.

(Participant 13)

#### 3.2.5. Theme 5: Protein Prioritisation

There is a focus on protein intake in female endurance athletes’ diets, which is particularly prioritised in snacks. There is an awareness that protein intake as a female athlete is important and should be a staple of meals and snacks to aid muscle repair and recovery.

I really, really need to think about increasing the amount of protein today (after a heavy/intense day) to reduce tired and heavy legs the next day, depending on what the session will be.

(Participant 13)

I’m pretty good at the protein snacks, I’ll do that throughout each day, its mostly the carb stuff I let slip.

(Participant 8)

Interestingly, food diary data suggests these perspectives appear to lead to adequate practices for protein intake (1.7 ± 0.7 g·kg^−1^ BM^−1^·d^−1^), with athletes achieving the current recommendations (1.2–2 g·kg^−1^ BM^−1^·d^−1^). Indeed, the inclusion of regular protein provision and protein snacks is something that athletes believe is very important and is also supported by the qualitative data, demonstrating protein intake throughout different meals (AM snack, 8 ± 7 g; Breakfast, 20 ± 10 g; Mid-morning snack, 12 ± 13 g; Lunch, 30 ± 12 g; PM snack, 13 ± 11 g; Dinner, 35 ± 20 g and evening snack, 7 ± 6 g of protein).

#### 3.2.6. Theme 6: Ageing

With the onset of age, female endurance athletes in this study appear to be more focused around the effect of nutrition on the body. However, some of the thoughts and perspectives of female endurance athletes are influenced by misinformation around human physiology. Whilst ageing may alter certain nutritional requirements [59,60,61,62,63], many athletes are fearful of age-related changes, which in some cases are not supported by science and based on misinformation.

I mean obviously my metabolism slowed down a lot. I mean I’m 47 so can tell I can’t get away with things anymore. I suppose it’s easy to put on weight.

(Participant 32)

From now on, thinking about things, I think well, I won’t have this or that, I don’t need as many carbs for where I am in my stage of life.

(Participant 2)

I suppose because I am coming from the mid 50 s, starting with the menopause, things like that I will notice my metabolism will slow down and joints become more stiff, so I need to avoid some inflammatory foods to help with things like that.

(Participant 2)

Thus, this theme demonstrates how ageing can shift athletes’ nutritional priorities, often toward health preservation and management. While some increased awareness of bodily changes with age is valid and supported by physiological evidence, many beliefs expressed by participants are rooted in oversimplified or inaccurate understandings of ageing and metabolism. Overall, the theme highlights a need for clearer education around nutrition for ageing athletes.

## 4. Combined Discussion

This study used quantitative and qualitative methods to explore female endurance athletes’ nutrition practices, influences, and perceptions. A key finding was the gap between female athletes’ nutritional knowledge and behaviour, particularly regarding CHO intake. Despite understanding its role in performance and recovery, athletes were unconsciously underfuelling with CHO. This behaviour appears to be influenced by factors such as personal relationships with food, time constraints, and “health halos”, a term used to describe misperceptions about the healthfulness of foods based on labelling or marketing [64].

Despite athletes’ awareness of the importance of CHO in their diet to support training, our data suggests that this does not translate into adequate CHO consumption. When comparing CHO intakes to the guidelines, even on days that are classified as ‘rest’, 50% of athletes adhere to the CHO guidelines, and on average, this amount equates to the lower end of the guideline range (3 g·kg^−1^ BM). The magnitude by which CHO practices fail to meet their respective guidelines increases from ‘moderate’ (−1.4 g·kg^−1^ BM), to ‘high’ (−3.5 g·kg^−1^ BM), to ‘very high’ (−5.5 g·kg^−1^ BM). This under-consumption can have significant implications for athletic performance, as inadequate CHO intake is associated with compromised glycogen replenishment, may impair muscle adaptation [65], and is linked with suboptimal recovery [66]. Chronic CHO inadequacy has been associated with disruptions in hormonal balance, bone health, metabolic function, immune response, and recovery, being a major risk factor [3,66,67]. In the present study, although total EI and energy derived from CHO increases as training volume and EEE increases, the magnitude by which EE exceeds EI is also further increased, suggesting that EEE is accompanied by increasing negative EB that does not appear to be compensated for through increased dietary CHO (nor any other macronutrient) intake. This gap between knowledge and practice aligns with previous research, which has highlighted similar trends in various athletic populations [2,8,68]. Previous research also suggests that while athletes may be educated on the benefits of CHO and recognise their importance, translating knowledge into dietary behaviour remains challenging [69,70,71,72]. The reasons for this discrepancy are likely multifaceted, including misconceptions about dietary needs, poor dietary planning, and possibly the influence of prevailing dietary trends that undervalue CHO consumption. The increasing energy deficit associated with higher training volumes observed in our study suggests that athletes are not adjusting their CHO intake to match their elevated EE, which is consistent with previous research indicating that athletes, particularly female athletes, are at risk of underfuelling relative to their energy needs [73,74], particularly in CHO intake [46,68]. The consequences of such deficits are potentially significant, such as being negatively linked with affecting performance, recovery, and overall health [75]. Historically, much of the work in relation to nutrition for female athletes surrounds the topic of LEA, a condition whereby insufficient energy is consumed to support the demands of exercise, potentially resulting in compromised physiological processes such as menstrual irregularities and impaired bone health [76,77]. The prevalence of LEA is reported as >60% in various female athlete groups [25,26,78,79]. More recently, the concept of LEA has been expanded into the RED-S model, which describes a broader spectrum of physiological and performance-related consequences. However, recent critiques highlight challenges in accurately measuring energy availability and isolating its direct effects from other influencing factors [25,80]. Many symptoms associated with RED-S, such as fatigue, impaired recovery, and metabolic disturbances, are multifaceted and may not always be solely attributable to LEA. Despite these complexities, prolonged or chronic periods of energy deficiency have been linked to negative health and performance outcomes. Regardless of terminologies or categorisation, the results of our study demonstrate that this energy gap is prevalent and a cause for concern. There is a clear need for more practical, hands-on education for athletes to bridge the gap between knowledge and application of CHO guidelines.

While athletes were found to have low CHO intake, protein consumption, in contrast, appeared to be more consciously prioritised. During interviews, protein was commonly referred to as the macronutrient athletes are most mindful of, supporting previous findings which report female athletes to perceive protein as critical for muscle repair and maintenance [81]. Our data suggest that this mindset leads to adequate protein intake with a mean intake of 1.7 g·kg^−1^ BM^−1^·d^−1^, thus falling comfortably within the recommendations for endurance athletes, 1.2–2 g·kg^−1^ BM^−1^·d^−1^ [10,16,82,83,84]. Previously, research documents varying protein intake patterns among female endurance athletes, with both adequate and inadequate protein intakes being reported [11,13], highlighting individual variability of protein intakes, likely influenced by training volume, dietary preferences, and nutritional knowledge. General recommendations for protein doses suggest that 0.25 g·kg^−1^ BM of a high-quality protein should be evenly distributed, every 3–4 h, across the day to maximise muscle protein synthesis (MPS) [10,85,86,87,88,89]. This generally matches athletes’ practices in the present study across the day. Interestingly, athletes in this study placed emphasis on high-protein snacks, which could be by virtue of increased availability of these items due to marketing and promotion of protein to consumers. The health halo effects from “protein”-labelled products have been found to be perceived as being healthier compared to control products without this label [90,91]. This cultural shift could therefore be influencing athletes’ perceptions and dietary behaviours, encouraging them to incorporate protein-rich foods and supplements to support their daily routines and athletic goals [84,92]. Whilst this appears to have a positive impact on protein habits, this prioritisation may impinge on CHO and overall EI. Protein is highly satiating [93], which could contribute to reduce total EI and the potential displacement of CHO, presenting a potential “catch-22” scenario. We highlight a potential role for promoting balance rather than avoidance, with practitioners encouraged to adopt simple, evidence-based strategies that pair CHO with protein, whether through specific recovery products, whole foods, or common options such as flavoured milk, protein smoothies, or yoghurt with granola, to optimise refuelling and recovery.

In this study, the athletes’ diets were influenced by several factors. Firstly, some athletes in the study reported negative attributions to body image, with a particular fear of future weight gain. A common theme amongst participants was the feeling of guilt associated with certain foods. Whilst it is not clear whether calorie deficits were driven intentionally, the qualitative data would suggest that these are largely unintentional, with most athletes stating the importance of adequate fuelling and reporting no intention to lose BM. The fear of weight gain observed aligns with the existing literature indicating that athletes, particularly women, are susceptible to body image concerns and weight-related anxieties. This is especially prevalent in sports that emphasise leanness or aesthetic appearance, where female athletes face unique pressures to maintain specific body weights or compositions [94,95]. Body weight is also often attributed to athletic success and personal value [96,97,98], which may be associated with more restrictive eating behaviours [99]. Such pressures contribute to a heightened risk of disordered eating and body image disturbances compared to non-athletes [100]. Whilst athletes in this study were not diagnosed with any eating disorders, many athletes exhibited traits of disordered eating, a subclinical condition composed of a range of irregular eating behaviours and negative body image. Participants would categorise foods as ‘good’ or ‘bad,’ which can result in feelings of guilt when ‘bad’ foods are consumed [101]. This dichotomous thinking not only fosters a negative relationship with food but also impairs nutritional adequacy [102].

Athletes’ behaviours are not only driven by personal perceptions but are also influenced by external factors such as coaching advice, societal norms, and the perceived ideals within their specific sport [96,103,104]. High levels of perfectionism and goal setting, often seen in athletes, can drive these perceptions and contribute to unrealistic and potentially unnecessary standards surrounding body image, leading to pressure, fear of weight gain, and lower self-esteem [105,106]. Positive reinforcement for weight loss or comments about body size can reinforce the belief that thinner is better [107]. Peer influence also plays a role; as seen in our data, athletes often compare themselves to their peers, leading to pressure to conform to perceived body norms within their sport [96].

Our findings underscore the considerable impact of others on athletes’ dietary practices, reflecting both challenges and opportunities for improvement. Many athletes reported conflicts stemming from the differing food preferences of partners or family members, which athletes reported to often make it more challenging to meet nutritional needs. This aligns with prior research showing that reliance on others for meal preparation, particularly in time-constrained athletes, can lead to suboptimal dietary choices and the phenomenon of dietary convergence, where athletes adjust their eating habits to match those around them, often to their detriment [108,109]. Such compromises not only impact nutrient intake but also contribute to feelings of guilt and frustration when athletes deviate from their intended practices. On the other hand, our findings highlight the importance of positive influences in shaping athletes’ nutrition. Supportive partners, family members, and qualified nutrition professionals were described by athletes as helpful influences that appeared to support healthier eating behaviours. Athletes described how professional guidance, such as tailored meal plans and education, improved their understanding of nutrition and helped them make informed decisions, consistent with previous research [110]. Supportive environments also alleviated time pressures and promoted adherence to dietary strategies, particularly for those with a negative history with food. These findings emphasise the need for evidence-based advice, as misinformation from social media continues to negatively influence athletes’ perceptions and behaviours [111,112]. Developing strategies to mitigate these external pressures and enhance access to reliable, science-based nutrition guidance is crucial for improving dietary practices. Beyond highlighting nutritional imbalances, these findings provide several applied implications for practitioners and identify key areas of potential intervention to be mindful of and potentially focus on. Time constraints and competing priorities repeatedly being identified as a barrier, suggests a need for simple, scalable meal-preparation strategies, for example, promoting batch cooking, ready-to-eat CHO options, or pre-prepared recovery snacks that align with athletes’ schedules. Furthermore, given the social influences observed, education for both athletes and their close networks (partners, coaches, teammates) may help create environments that normalise flexible and supportive fuelling practices. Integrating behavioural strategies, such as planning CHO–protein combinations post-exercise and using visual or app-based reminders, could help translate knowledge into consistent practice. Collectively, these insights highlight practical touchpoints where practitioners can direct future interventions to promote balanced, accessible, and contextually appropriate fuelling behaviours, rather than prescriptive or restrictive approaches.

## 5. Limitations

This study highlights participant compliance and challenges with food photography methods. Burrows et al. [113] noted that participant burden affects compliance and data quality, while Martin et al. [31] found that photo quality (lighting, resolution, angle) impacts reliability. Estimating portion sizes introduces variability and potential errors [114]. To address these issues, athletes in this study received training, weighed all food and drink items alongside photos, and could also use audio descriptions for efficiency and context. High-quality food diaries were combined with interviews for a broader understanding of nutritional behaviours. Participants could choose any 4-day period, including optional weekend days, to enhance inclusion and adherence, with weekday/weekend patterns analysed where relevant.

While individual cycling efficiency can vary between approximately 18–25%, sensitivity analyses showed that this had minimal impact on the overall relationships between EEE and EI. However, small shifts in estimated EEE (~<100 kcal·day^−1^ across plausible GE ranges) may still represent meaningful discrepancies at the individual level, particularly in applied monitoring of energy availability. This reinforces the importance of combining standardised analytical approaches with practical, athlete-specific interpretation when evaluating fuelling adequacy.

Estimating EEE from HR data carries several limitations that should be acknowledged. Although chest- and arm-based HR monitors (e.g., Polar, Garmin, COROS) provide relatively accurate HR measurements, variability in the HR–VO_2_ relationship across individuals, environmental influences, and day-to-day physiological variation can affect precision. HR-based algorithms may overestimate EE during non-steady-state exercise or underestimate it during high-intensity bouts due to delayed HR kinetics. Moreover, these methods assume a fixed linear HR–VO_2_ relationship, which may not hold across all intensities or modalities. While the average estimation error is expected to fall within ±10–15%, small inaccuracies could accumulate when interpreting day-level EB data [39,40,41,42]. Nevertheless, this approach provides a practical and validated method for quantifying EEE across diverse endurance training modalities in field settings.

It is acknowledged that participants self-selected the 4-day recording period, which may introduce bias toward reporting days perceived as more representative or favourable in terms of dietary habits. Although participants were encouraged to include at least one weekend day to capture the variability typically seen in training and nutrition patterns, self-selection was included to optimise feasibility and compliance given participants’ diverse work, family, and training commitments. Weekend days often constitute higher training volumes and therefore greater nutritional challenges; consequently, if underfuelling was observed despite self-selection, the true magnitude of this issue may be even more pronounced across the broader training week. Additionally, self-report methods carry the possibility of under-reporting, particularly on high-training-load days when time constraints and fatigue may reduce accuracy, representing a potential confounding factor that should be considered when interpreting the observed associations. Lastly, as daily entries were analysed independently, it is possible that some within-participant correlation exists. However, the study objective was not to model change over time, but to describe observational day-level relationships between EE and intake.

## 6. Conclusions

This study aimed to understand the nutrition practices, habits, and perceptions of female endurance athletes using a mixed methods approach. Findings revealed that while protein intake was generally adequate, CHO intake often fell short, particularly on high-volume training days. Athletes’ underfuelling appeared to be unintended and shaped by complex behaviours that warrant further research. Positive social influences, like supportive relationships and nutritionists, were linked to better adherence to nutritional guidelines, emphasising the importance of a supportive environments and informed advice. The study highlights the interplay between nutrition, psychological factors, social influences, and athletic performance, stressing the need for personalised interventions to optimise dietary practices, improve performance, and promote long-term health. These findings also suggest that nutritionists could benefit from biopsychosocial education and training, to better address the multifaceted needs of female endurance athletes. Future research should also explore how competitive environments influence nutrition behaviours and how applied interventions can most effectively support athletes across diverse real-world contexts.

## Figures and Tables

**Table 1:** Participant characteristics as means and standard deviations (SD), age ranges, and weekly training volume (*n* = 72).

**Participant Characteristics**	
Age (years)	42 ± 9
Body mass (kg)	61.7 ± 8.8
Height (cm)	1.6 ± 0.1
**Age Range (Years)**	
18–29	9
30–39	23
40–49	25
50–59	14
>60	1
**Training Volume (h·week^−1^)**	
7–9	29
10–12	20
13–15	12
>15	11

**Table 2:** Total, relative, and percentage contribution of macronutrients, means ± SD.

Variable	CHO	Fat	Pro
Total (g)	233.1 ± 94.2	79 ± 31	105 ± 38
Relative (g·kg^−1^)	3.8 ± 1.6	1.3 ± 0.5	1.7 ± 0.7
Contribution (%)	43.1 ± 11.0	33.3 ± 10.1	20.4 ± 6.4

**Table 3:** Average total energy intake, energy expenditure, energy balance, and accumulative energy balance, means ± SD.

Variable	Mean ± SD
EI (kcal·day^−1^)	2107.7 ± 611.1
EE (kcal·day^−1^)	2289.0 ± 557.1
EB (kcal·day^−1^)	−193.4 ± 576.3
4-day EB (kcal)	−772.1 ± 1523.3

**Table 4:** Energy balance across different days of the week, weekday vs. weekend, presented as means ± SD.

**Day**	**EB (kcal·day** **^−^** **^1^** **)**
Monday	−3.8 ± 396.0
Tuesday	−56.6 ± 522.5
Wednesday	−132.2 ± 571.7
Thursday	−231.2 ± 480.4
Friday	−202.9 ± 458.9
Saturday	−474.2 ± 834.7
Sunday	−335.6 ± 579.9
**Period**	**EB (kcal·day** **^−^** **^1^** **)**
Weekdays	−119.4 ± 499.5
Weekend	−420.3 ± 743.1

**Table 5:** Energy balance across different age categories, means ± SD.

Age Category (Years)	*n*	EB (kcal·day^−1^)
18–29	9	−482.2 ± 71.0
30–39	23	−182.3 ± 482.3
40–49	25	−174.0 ± 349.4
50–59	14	−120.1 ± 217.1
≥60	1	−768.4

**Table 6:** Summary of themes with corresponding participant distribution, age range, training volume, and primary sport discipline. Participants could contribute to multiple themes due to overlapping experiences.

Theme	Brief Description	*n*	Age Range (Years)	Training Volume (h·week^−1^)
Unintentional underfuelling	Gap between nutritional knowledge and habitual fuelling	14	24–57	7–15
Negative relationship with food	Guilt, body image concerns, restrictive eating	9	24–37	9–15
Relationships and other influences	Social, family, and professional influences	9	24–38	7–12
Perceived lack of time	Time pressure and convenience-driven choices	10	27–52	7–15
Protein prioritisation	Emphasis on recovery foods and protein intake	12	24–57	9–15
Ageing	Menopause, ageing beliefs, metabolic perceptions	6	45–57	7–12
Total		19	24–57	7–15

## Data Availability

Data supporting the findings of this study are available from the corresponding author upon reasonable request.

## Abbreviations

The following abbreviations are used in this manuscript:

CHO	Carbohydrate
LEA	Low energy availability
RED-S	Relative energy deficiency in sport
EEE	Exercise energy expenditure
EE	Energy expenditure
EI	Energy intake
EB	Energy balance
GE	Gross efficiency
HR	Heart rate
BM	Body mass
